# Numerical Investigation on Lateral Confinement Effects on Concrete Cracking Induced by Rebar Corrosion

**DOI:** 10.3390/ma13051156

**Published:** 2020-03-05

**Authors:** Gyeongcheol Choe, Yasuji Shinohara, Gyuyong Kim, Jeongsoo Nam

**Affiliations:** 1 Department of Architectural Engineering, Chungnam National University, 99 Daehak-ro, Yuseong-gu, Daejeon 34134, Korea; choegc@cnu.ac.kr (G.C.); gyuyongkim@cnu.ac.kr (G.K.); 2 Laboratory for Future Interdisciplinary Research of Science and Technology, Tokyo Institute of Technology, Yokohama 226-8503, Japan; yshinohara@ca.em-net.ne.jp

**Keywords:** accelerated corrosion test, reinforced concrete, corrosion, crack behavior, finite element analysis

## Abstract

Accelerated corrosion tests of reinforced concrete (RC) specimens were conducted to estimate the corrosion expansion rate of reinforcing bars. Subsequently, finite element analysis was performed with the estimated expansion rate for RC beams to investigate concrete cracking induced by corrosion. The influence of the different confinement levels on crack behavior was investigated using mainly the amount of transverse reinforcement. An expansion rate of 2 was found to be appropriate when using Lundgren’s expansion model. Confinement levels affected the cracking behavior of steel bars. Cracks did not significantly affect structural capacity although they exceeded the allowable crack width. Nevertheless, repair and reinforcement measures are necessary because degrading durability factors such as carbonation or salt diffusion can reach the reinforcing bars through connected cracks.

## 1. Introduction

The design of reinforced concrete (RC) structures requires the consideration of durability in terms of sustainable use, as well as maximum strength for safe structural performance. During the service life of structures, RC members are affected by several environmental factors, such as carbon dioxide or chloride penetration [1,2,3]. Therefore, steel corrosion has a significant influence on the serviceability and durability of RC structures [4,5,6,7]. Generally, steel corrosion leads to critical deterioration, such as cracking or cover spalling of RC structures, because corrosion products occupy more than 2–6 times the volume of the original steel [8]. In particular, steel corrosion can result in pressure expansion, leading to tensile stress in the concrete surrounding the steel bars [7,9]. Cracking occurs when the tensile stress exceeds the tensile strength of the concrete. concrete cracking induced by corrosion in RC has a significant impact on the serviceability and durability of RC structures, which may cause structural damage such as the reduction of bond strength and the spalling of concrete cover. It is therefore very important to investigate concrete cracking induced by corrosion in order to achieve suitable maintenance control for RC structures [8,9,10].

Wide attention has been paid to the modeling of cracking behavior under reinforcement corrosion in RC structures [2,3,4,5,6,7,8,9,10,11,12,13,14,15,16,17,18,19,20,21,22,23]. Liu and Weyers [3] proposed a corrosion cracking model able to predict the time-to-corrosion cracking of concrete cover. The time-to-corrosion cracking was experimentally investigated using the results of cracked RC slabs under 5 years of outdoor exposure, during which the effects of corrosion rate, concrete cover depth, reinforcing steel bar spacing, and size were considered. They found that concrete cracking was affected by the critical weight of the corrosion products depending on the cover depth, the bond performance between the steel bars and concrete, and the size of the steel bars. Moreover, Bhargava et al. [7,10,11] developed a mathematical model for predicting the time-to-corrosion cracking of concrete cover as well as the weight loss of steel bars. According to the results of their study, the tensile strength of concrete cover, the corrosion rate, and the elastic modulus of steel bars with corrosion products have a significant effect on the models for time-to-corrosion cracking of concrete cover and for the weight loss of steel bars. In addition, the concrete cracking induced by corrosion has been widely investigated through experimental and numerical studies, focusing on the influences of corrosion expansion pressure on RC [24,25,26,27,28,29,30,31,32,33,34,35,36,37,38,39,40]. Expansion pressure is caused by expanding corrosion products which have grown around steel bars, which is also a primary factor for the cracking behavior. These previous works provide critical and useful information for predicting the concrete cracking induced by corrosion of RC structures. However, they did not consider the lateral confinement effect in RC structures. It is necessary to consider lateral confinement for RC structures as well as for longitudinal bars, which may affect the cracking of concrete cover under natural environmental conditions. 

Lin and Zhao [41] investigated the effects of confinement on the bond strength between concrete and corroded steel bars. They found that the bond strength of corroded steel bars was significantly increased by confinement effect of the ties, which also contributed to limiting the longitudinal cracking of concrete cover. Since the expansion coefficients depend on the service environment, the precise evaluation of their values in the considered environment is necessary for the application of a suitable model of concrete cracking induced by corrosion in RC structures [5]. Fang et al. [42,43,44] also reported the effects of confinement on the bond behavior of RC structures under the salty environment of corrosion. The bond performance did not decrease in cases of confinement for stirrups compared to those without the confinement of RC structures. This means that confinement greatly contributes to resistance against deterioration caused by corrosion expansion of the steel bars. Corrosion expansion is remarkably controlled by the characteristics of corrosion products and by the actual confinement condition of the surrounding corroded steel bars. The actual confinement in RC structures is given by transverse bars and the surrounding concrete [34,45,46].

As described above, the effect of lateral confinement of transverse reinforcement on the concrete cracking induced by corrosion of RC structures still needs to be explored further. Therefore, this study aimed to investigate the effect of lateral confinement on the cracking behavior of RC structures caused by the corrosion expansion of steel bars. The results may provide useful information for determining the relationship between a given corrosion level of steel bars and the structural performance of RC structures. In order to propose an appropriate corrosion expansion ratio, an experimental work was performed using accelerated electrolytic corrosion tests, in which a volume expansion model of corrosion products required for analyzing the cracking behavior was used. The concrete cracking induced by corrosion of RC beams was studied numerically using the corrosion expansion ratio obtained from an experimental program. In particular, the main parameters of this numerical simulation are the lateral confinement by transverse reinforcement, position of steel bars, radial direction of expansion, and loading method controlled by pressure expansion. Moreover, the relationship between crack propagation and the amount of reinforcing steel corrosion (corrosion penetration and partial loss) is discussed using the expansion coefficient and mechanical properties of corrosion products. Finally, the suitability of the current numerical study is examined by comparing the results with reference experimental data on corrosion cracking.

## 2. Expansion Model of Steel Corrosion Products

**Figure 1:**
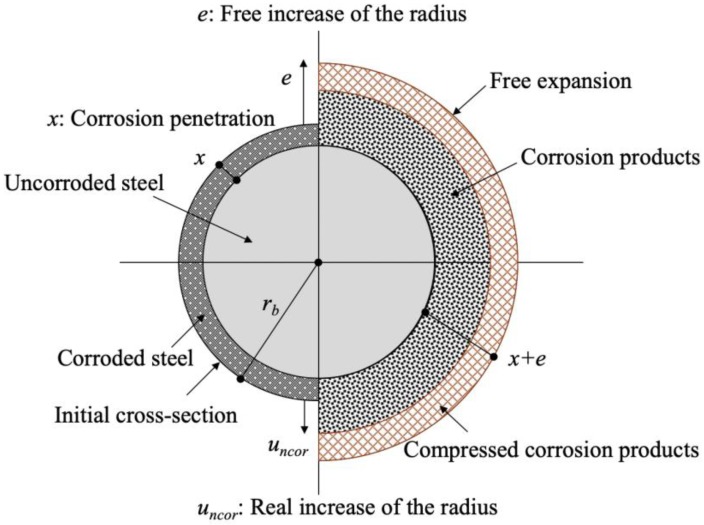
Schematic of the corrosion model for volume increase with corrosion products [18].

**Figure 2:**
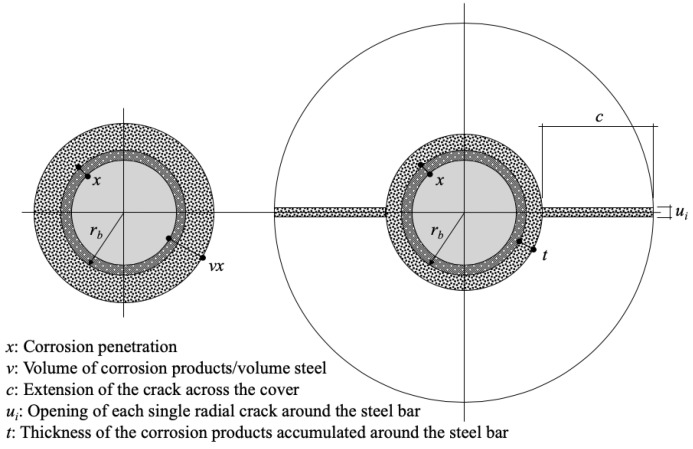
Schematic of the corrosion model for corrosion penetration and the corrosion products around the steel bar [47].

Concrete cracking induced by corrosion occurs when the volume of corrosion is larger than the original volume of corroded steel bars. Lundgren [18] and Berra et al. [47] proposed a corrosion expansion model for the corrosion layer of steel bars by comparing the volume increase of corrosion products (rust) and virgin steel. Figure 1 shows the corrosion expansion model proposed by Lundgren. In the figure, all corrosion products accumulate around the steel bar, and the corrosion products do not penetrate voids and cracks in concrete materials. This model may correspond to a relatively short period of time-to-corrosion, such as an electrolytic corrosion test. Berra et al. also proposed a model in which corrosion products penetrate voids and cracks in concrete, which corresponds to the progression of corrosion over a long period of time, as shown in Figure 2. In natural environments, the behavior of corrosion products can be postulated to lie in between the Lundgren and Berra et al. models.

According to Lundgren’s model [18], by assuming that the volume of the corrosion products is $v_{0}$ times the volume of the virgin steel bar, under a given corrosion penetration *x* (mm), the free increase of the radius *e* (mm) is determined using Equation (1) from the geometric relationship

(1) $$e=-r_{b}+\sqrt{r_{b}{}^{2}+\left(v_{0}-1\right)\left(2r_{b}x-x^{2}\right)}$$

By assuming that the corrosion penetration *x* is sufficiently small relative to the radius of a steel bar *r_b_* (mm), the free increase of the radius *e* can be expressed as

(2) $$e\approx \left(v_{0}-1\right)x$$

Meanwhile, according to Berra’s model [47], after ignoring the elastic modulus of concrete, and by assuming that the increased circumference due to expansion is converted into a crack width, the sum of all the cracks opening around a bar is

(3) $$w_{cr}=\sum u_{i}=2\pi (r_{b}+t)-2\pi r_{b}=2\pi t$$

where *w_cr_* is the total crack width (mm), *u_i_* is the opening of each radial crack width around a steel bar (mm), *r_b_* is the radius of a steel bar, and *t* is the thickness of corrosion products that have accumulated around a steel bar.

**Figure 3:**
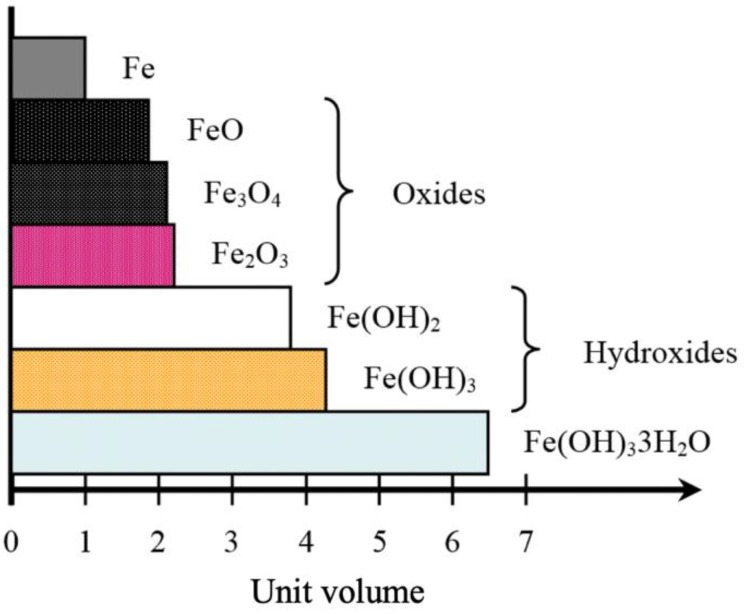
Relative volumes of iron corrosion products [18,20,24].

Additionally, by assuming that the corrosion products completely penetrate into cracks, *t* can be expressed as

(4) $$2\pi r_{b}vx=2\pi r_{b}(x+t)+2\pi tc\Rightarrow t=\frac{r_{b}(v-1)}{r_{b}+c}x$$

where *v* is the volume of corrosion products/volume steel, *x* is the corrosion penetration (mm), and *c* is the extension of the crack across the cover (mm). In Lundgren’s model, assuming that the extension of the crack across the cover *c* is 0, the thickness of the corrosion products that have accumulated around the steel bar *t* matches the free increase of the radius *a*. The ratio *v* between the volume of the corroded and virgin steel varies depending on the composition of corrosion products. Various rust products have different densities and volume expansions; the values of *v* for corrosion products vary from 2.2 to 6.4 [18,20,24], as shown in Figure 3. It is expected that crack initiation and propagation are directly proportional to the amount of corrosion products. In this study, because the diffusion of corrosion products into pores and cracks is not taken into account in Lundgren’s model, the value of 2.0 was considered for the numerical analyses of the concrete cracking induced by corrosion of RC Beams with reference to Lundgren’s model.

In Equation (1), *e* is the increase of the radius due to free expansion when the normal stress is zero. However, when the corrosion products are restrained by the concrete surrounding the steel bar, they cannot expand freely. The actual increase of the radius, *u_cor_*, is obtained from the strain, *ε_cor_*, of rust by the equation

(5) $$\varepsilon_{cor}=\frac{u_{cor}\;-\;e}{x\;+\;e}$$

**Figure 4:**
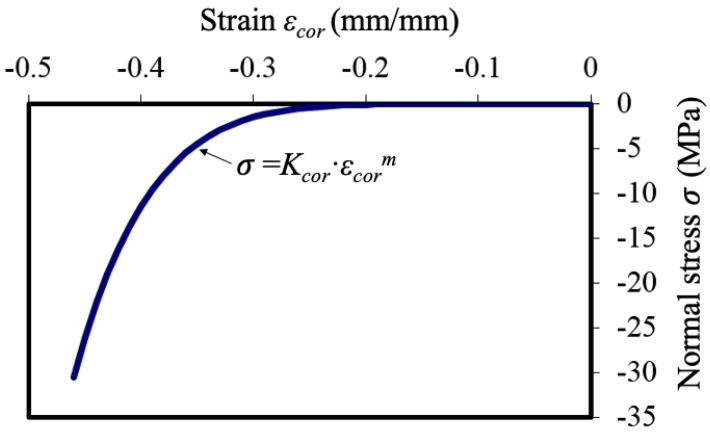
Relationship between the normal stress and strain of corrosion products [18].

Lundgren [18] proposed a non-linear equation as a relationship between the normal stress, σ, developing around rebar, and the corresponding radial strain, *ε_cor_*, in the rust on the basis of experimental data, as shown in Figure 4 and Equation (6).

(6) $$\sigma =K_{cor}\times \varepsilon_{cor}{}^{m}\;K_{cor}=0.7GPa,\;\;m=0.7$$

## 3. Accelerated Corrosion

### 3.1. Specimens

The composition and characteristics of concrete used in the current study are listed in Table 1. For the concrete mixture, type II Ordinary Portland cement, fine aggregate (river sand) with a grain size of less than 5 mm, and coarse aggregate with a maximum size of 20 mm were used. A water-to-cement ratio (W/C) of 0.5 was applied, and a water reducer of 0.8% (wt % by cement) was added to achieve proper workability. In accordance with ASTM C39 [48], the compressive strength was evaluated using a universal testing machine (UTM). All compression tests were performed using cylindrical concrete specimens with diameters of 100 mm and heights of 200 mm. The specimens for the compression tests were cured in water at a temperature of 20 ± 2 °C for 28 days. The compressive strength of the concrete cylinder at 28 days was 42.5 MPa, which is the average value of three tests.

### 3.2. Test Method

**Figure 5:**
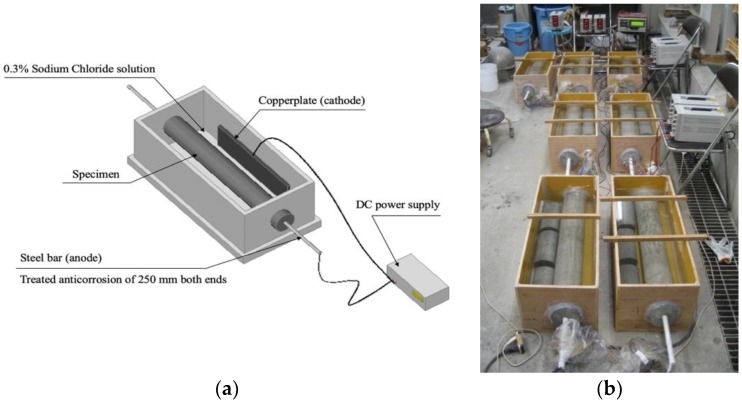
Accelerated corrosion system: (**a**) schematic of accelerated corrosion tests; (**b**) specimens under accelerated corrosion.

**Figure 6:**
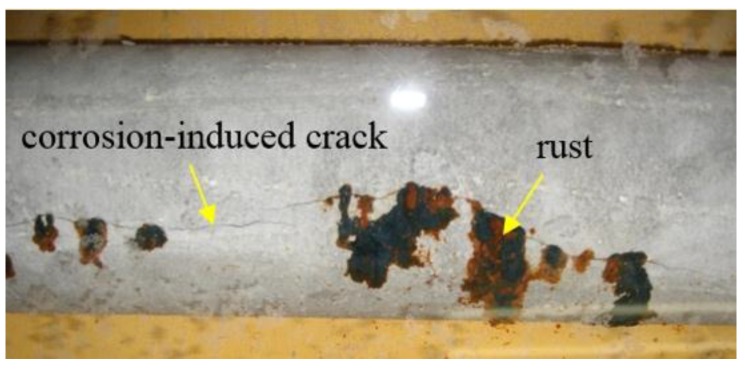
Photograph of typical cracks and rust in RC specimens after accelerated corrosion tests.

**Figure 7:**
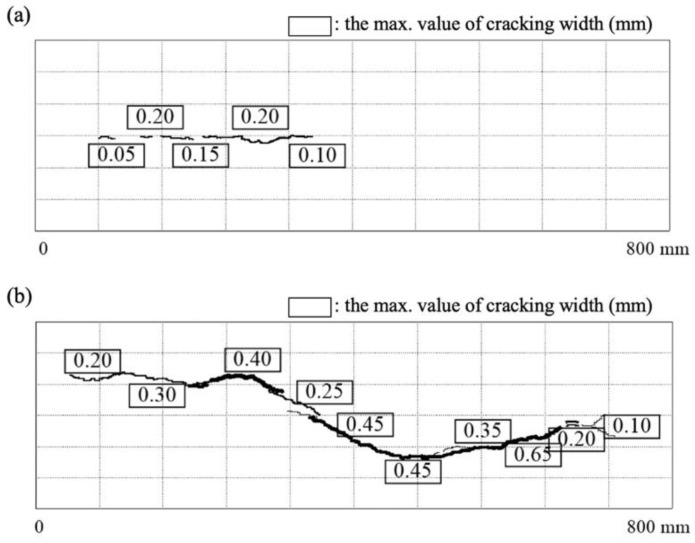
Cracking propagation due to corrosion expansion of steel bars: (**a**) specimen A with corroded steel ratio of 2.72%; (**b**) specimen B with corroded steel ratio of 3.95%.

Cylindrical specimens that were 125 mm in diameter and 800 mm in length were fabricated for accelerated corrosion tests. A deformed reinforcing steel bar with a diameter of 19 mm and a length of 1200 mm was embedded in the center of the specimen. Anticorrosion treatment was applied over a length of 250 mm from each end of the steel bar, leaving a 700-mm corrosion section of the steel bar set between the two ends. Figure 5 shows the accelerated corrosion system of RC specimens. Accelerated corrosion tests of the reinforcing steel were conducted with energized electric current of 200 mA (current density 0.48 mA/cm^2^) until cracks appeared on the concrete surface. Figure 6 shows the typical condition for cracking and rust formation in RC specimens after accelerated corrosion tests. Figure 7 additionally shows the cracking propagation behavior of the steel bars due to corrosion expansion. Specimen A represents cracking properties after 337 h from the start of the application of electric current. Specimen B represents cracking properties after continuing the current supply to a total of 450 h. From the figures, concrete cracking induced by corrosion was generated from only one main-line in RC specimens, even with sustained energized outflow of corrosion products through the cracks. The numbers in boxes in Figure 7 show the crack width (mm), and the corrosion amount was defined in terms of mass loss by measuring the mass of specimens before and after corrosion.

## 4. Axial Symmetric Simulation of the Corrosion Expansion

**Figure 8:**
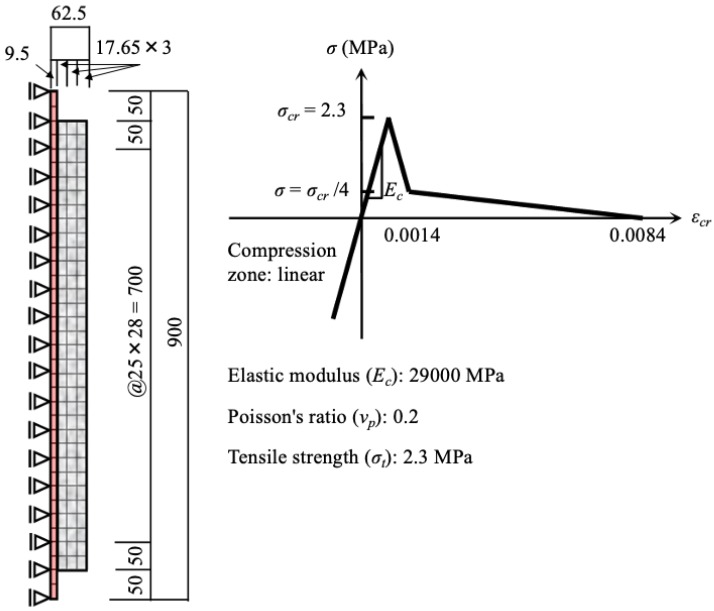
Axial symmetry model and mechanical properties of concrete.

Considering the amount of corrosion and corrosion inflation pressure at the time when corrosion expansion cracking reached the surface, the same mechanics state was assumed for the cross-section through the central axis until the occurrence of cracks. An axial symmetric model was used in the corrosion expansion simulation, as shown in Figure 8. Finite element analysis was conducted by using the program DIANA [49]. Loading control was performed such that the 700-mm corrosion zone of the concrete imposed a pressure increment of 0.5 MPa, with further increments of 0.1 MPa of pressure being applied after the pressure of 11 MPa as cracks approached the surface of the RC specimens. Bilinear-type tension softening properties of concrete were used to reflect the actual characteristics because there was no restraint bar for cracks. In the present analysis, the compression area was assumed to exhibit elastic behavior because tensile cracking was dominant under low compression stress. The start of the cracks was defined according to the maximum principal stress criterion of Rankine [50]. Namely, when the maximum principal stress reaches the tensile strength of concrete, regardless of the stresses acting on the other side, cracks are formed perpendicular to the direction of the maximum principal stress. Thus, the multi-directional dispersion cracking model (threshold value of 60°) was used.

**Figure 9:**
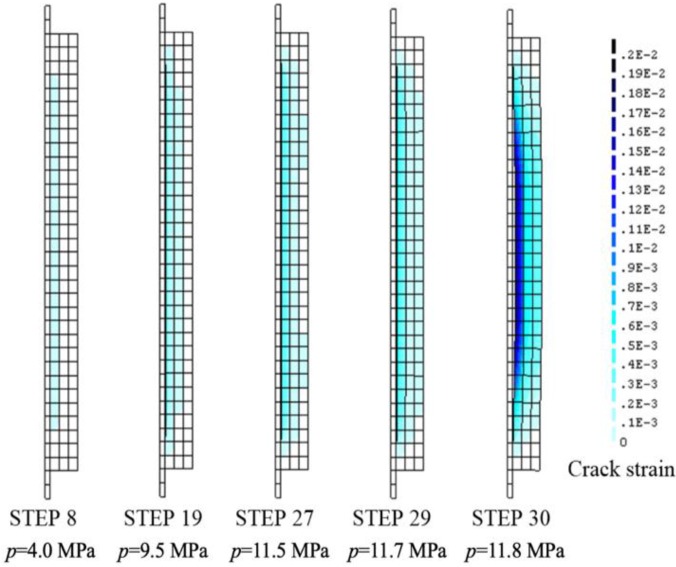
Crack strain contour and deformation properties.

In this study, a simplified version of Lundgren’s model is used because evaluating corrosion products entering the cracks of the concrete is difficult. Therefore, the value of 2 was used for the corrosion expansion ratio *v*. Figure 9 shows the typical crack strain contour and deformation properties according to the incremented load. In addition, the results of corrosion expansion properties for each step are listed in Table 2. Concrete cracking was generated at the inner integration point of the innermost layer under an expansion pressure of 4.0 MPa and reached the second layer under an expansion pressure of 9.5 MPa. Moreover, concrete cracking proceeded to the inner integration point of the outermost layer under an expansion pressure of 11.7 MPa, but in this state, cracks did not appear on the concrete surface, and deformation could not be confirmed even at 200 times magnification. Concrete cracking developed up to the outer integration point of the outermost layer at an expansion pressure of 11.8 MPa, which was equivalent to the amount of corrosion. For step 30, the expansion radius was observed to increase rapidly, and deformation could be observed at 200 times magnification. As corrosion products were discharged through the cracks in the experiment, the pressure increment was not necessarily sustained. Nevertheless, in the numerical simulation (corrosion amount of 2.94%), an expansion pressure of 11.8 MPa corresponded well to the experimental results (corrosion amount of 2.72%). Surface cracking was confirmed. As the numerical model used in this study is an axial symmetry model, strain by cracking in the outer integration point of the outermost layer occurred uniformly in the circumferential direction. From the numerical results, crack width could be evaluated by multiplying the circumferential length by the strain in the outer integration point of the outermost layer. Cracking appeared on the surface (expansion pressure of 11.8 MPa) with a maximum crack strain, *ε_cr max_*, of 2.95 × 10^−4^, and a circumferential length of 393 mm. Thus, crack width was calculated to be 0.12 mm. The crack width determined from the numerical results may be considered a reasonable value because it lies within the range of 0.05–0.2 mm in width experimentally measured from the cracking of specimen A. Therefore, when performing corrosion expansion using Lundgren’s model, the corrosion expansion ratio of 2 is reasonable.

## 5. Corrosion Expansion in the Numerical Model and Material Properties of RC Beams

**Figure 10:**
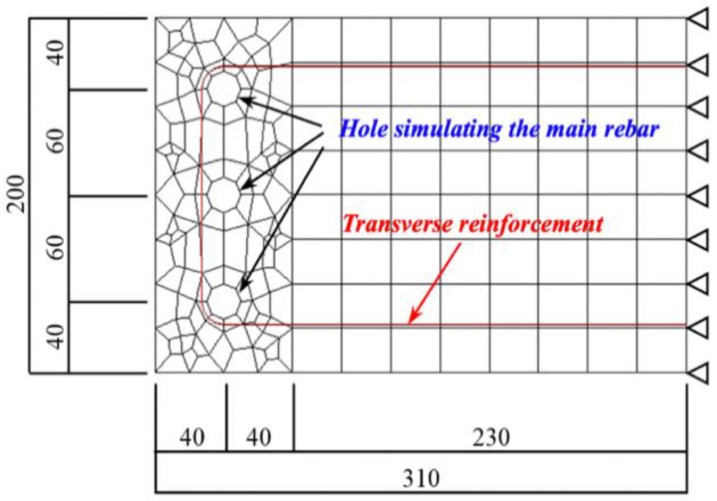
Numerical model and boundary conditions.

Figure 10 shows the numerical model of corrosion expansion for RC beams. In this numerical model, the main rebar was the target of corrosion, and transverse reinforcement (shear reinforcement) was applied to restrain the expansion cracks. For concrete, two-dimensional plane stress elements with a transverse reinforcement spacing of 60 mm were applied. A 20-mm diameter hole was assumed for the main rebar, and embedded steel elements with three levels of cross-sectional area (0, 28, 112 mm^2^, corresponding to shear reinforcement ratios *p_w_* = 0, 0.47, 1.87%) were assumed for transverse reinforcement. Based on the previous experiment, the corrosion expansion ratio *ν* of 2 was used.

**Figure 11:**
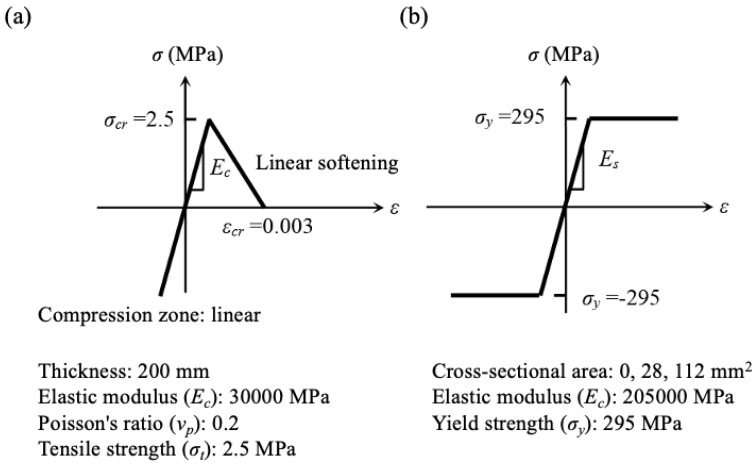
Stress–strain curve and mechanical properties of elements used in the numerical model: (**a**) concrete; (**b**) transverse reinforcement.

The stress–strain curve and mechanical properties of concrete and transverse reinforcement used in the numerical simulation are shown in Figure 11. As mentioned earlier, because tension cracking of concrete was dominant under low compressive stress, the maximum principal stress criterion of Rankine was applied to the tension zone, and elastic behavior was assumed in the compression zone. As shown in Figure 11, a linear model with a fracture energy of 0.1 N/mm (characteristic length of the element is 27 mm) was used for tension-softening behavior after cracking. In addition, shear stiffness after cracking decreased in accordance with the crack strain. A bi-linear model of transverse reinforcement (no strain hardening behavior) with standard yield strength was applied. The pressure buildup around the corroded bar was applied in 0.1 MPa increments using a loading method with an internal pressure control. Additionally, the right end of the numerical models was controlled with a pinned support, because the support condition does not affect the cracking behavior. Table 3 shows details of specimens used in the numerical simulation. 

## 6. Results and Discussion

### 6.1. Cracking Behavior Due to Corrosion Expansion

**Figure 12:**
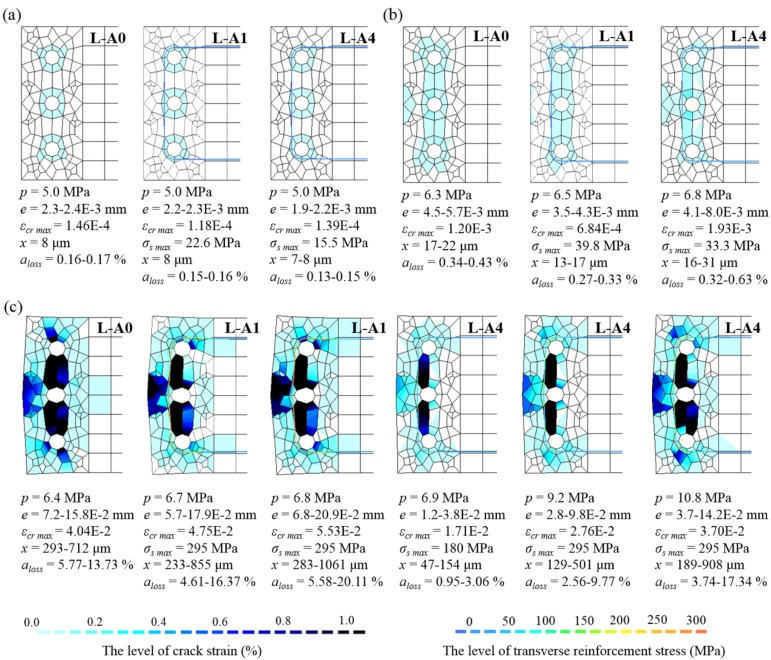
Comparisons of concrete cracking induced by corrosion at each step: (**a**) early corrosion; (**b**) cracking reaching the surface of concrete; (**c**) final cracking progress.

Figure 12 shows the typical concrete cracking induced by corrosion and transverse reinforcement stress for each step of corrosion. The pressure exerted by corrosion products on the surrounding concrete, *p*, corrosion expansion (increase in radius), *e*, maximum crack strain, *ε_cr max_*, and transverse reinforcement stress, *σ_s max_* are also shown in these figures. Furthermore, the corrosion penetration, *x*, and the reduction of the cross-sectional area of main bars, *a_loss_*, which were estimated using the method described in the previous section, were added to the corresponding loading steps. In the cases of each crack progressing independently, similar concrete cracking induced by corrosion was observed in RC specimens regardless of the mass of transverse reinforcement. However, as shown in Figure 12b, if developing cracks connect with each other and reach the surface of concrete, then the pressure–expansion relationship and crack behavior will depend on the amount of transverse reinforcement. After the crack reached the surface of concrete, corrosion penetrations were 20–30 μm, and differed slightly among specimens and according to the rebar location. Considering that the cross-section loss was less than 1.0% after the crack reached the surface of concrete, it can be considered to have almost no effect on the ultimate strength of the concrete as a structural member. In general, reaching the service limit is defined by a crack opening at the surface of concrete in RC structures. Nevertheless, when the cracking reaches the surface of concrete, the reduction in structural capacity is small, and thus, the service limit is a passive standard from the perspective of structural performance [29,34]. However, if the cracks spread across the surface of concrete, the risk of carbonation and salt damage can be increased.

In cases of final cracking progress (Figure 12c), for the L-A0 specimen without transverse reinforcement, the concrete cover spalled when a crack reached the surface of the concrete cover and it could not resist the increasing pressure. The spalling was controlled for the L-A1 specimen immediately after the crack reached the surface, but deformation increased rapidly after the transverse reinforcement failed. In addition, the maximum crack strain of the L-A1 specimen exceeded 0.04, with a crack width greater than 2 mm and a crack spacing of 50 mm. Thus, spalling and adhesion degradation of the concrete cover are expected. For the L-A4 specimen with four times the mass of transverse reinforcement compared to that of the L-A1 specimen, deformation was considerably less even under high pressures because of the strong confinement. Corrosion penetration and cross-section loss during the final cracking progress were 300–1000 μm and 5–20%, respectively. Additionally, the resistance to expansion due to corrosion reached its limit after transverse reinforcement members yielded. The analytical results obtained from displacement-controlled experiments show generally similar cracking behavior and pressure–expansion relationships regardless of the amount of transverse reinforcement because it only adds a little stiffness to the concrete. The expansion behavior of corrosion products in an actual RC structure is close to the results of the pressure-controlled experiments, as some amount of corrosion products is lost through cracking and spalling.

### 6.2. Internal Pressure and Expansion by Corrosion of Steel Bar 

**Figure 13:**
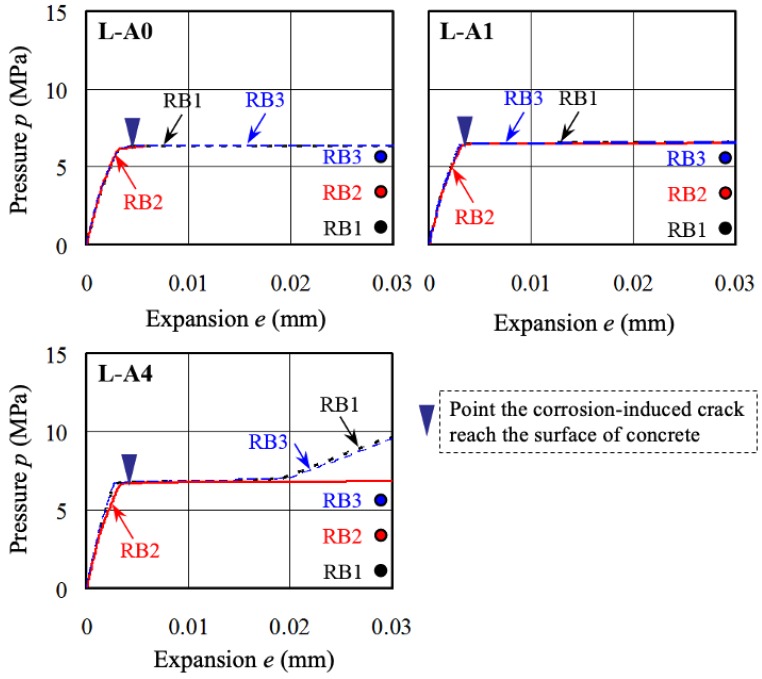
Relationship between average internal pressure and expansion according to the location of steel bars for each specimen.

Figure 13 shows the relationship between average internal pressure and expansion according to the location of the steel bar for each specimen. In the figure, RB 1, RB 2, and RB 3 are the low bar, middle bar, and upper bar among the main reinforcement members, respectively. For corrosion expansion up to about 0.002–0.003 mm, the average internal pressure of each specimen almost completely corresponded to cracks that appeared independently around each longitudinal bar. In addition, the average internal pressures of RB 1 and RB 3 exhibited similar behaviors, which is expected because their symmetrical positions. When cracks reached the surface of the concrete at internal pressures *p* of 6–7 MPa (corrosion penetrations of 20–30 μm), corrosion expansion progressed rapidly because the resistance against expansion depends only on the resistance performance of transverse bars, rather than concrete. In cases of the L-A4, resistance against expansion became effective after the corrosion expansion exceeded a value of 0.02 mm.

**Figure 14:**
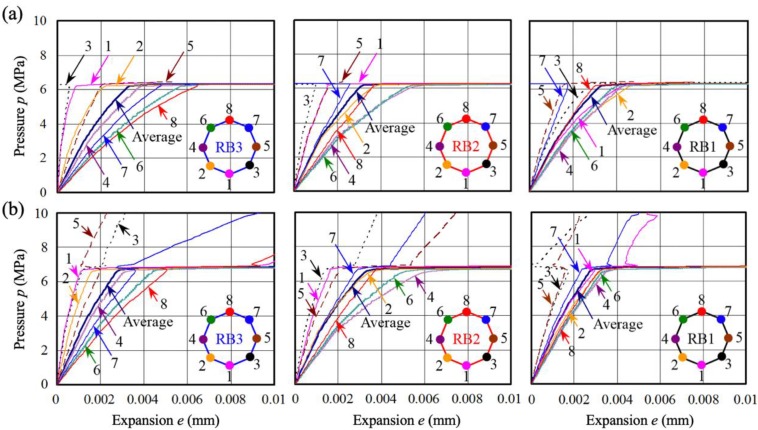
Relationship between internal pressure and expansion according to the expansion direction of longitudinal bars: (**a**) L-A0, without lateral confinement; (**b**) L-A4, with large lateral confinement.

Figure 14 shows the relationship between internal pressure and expansion (increase in radius) according to eight directions of longitudinal bars with and without lateral confinement. When cracks reached the surface of the concrete at internal pressures *p* of 6–7 MPa, the stiffness of the concrete was almost eliminated. However, stiffness was found to vary depending on the direction of expansion. Generally, stiffness against corrosion expansion decreases as progress is made in the direction of lower cover thickness. Nevertheless, the figure shows that the stiffness increased with the addition of lateral confinement members (directions of 1, 2, 3, 4, and 6 in RB 1; and 2, 4, 6, 7, and 8 in RB 3). Meanwhile, the stiffness of RB 2 was not improved in the direction of point 4 because the transverse bars did not function effectively for the expansion on the left side. Thus, in order to ensure stiffness in the direction of point 4, additional internal rebars are required. Along directions corresponding to high stiffness, such as 3, 7, and 8 of RB 1, and 1 of RB 3, deformation occurred with rapid expansion due to internal pressure from the opposite direction.

### 6.3. Maximum Stress in Transverse Bar and Corrosion Penetration

**Figure 15:**
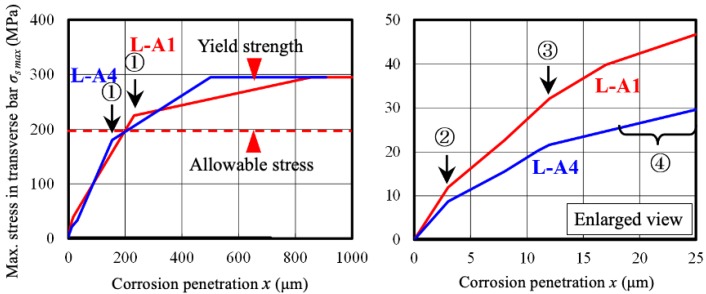
Relationship between maximum stress in transverse bars and corrosion penetration.

Figure 15 shows the relationship between maximum stress in transverse bars and corrosion penetration. The right figure is an enlarged drawing for a small corrosion level. At point ①, where the cracks spread across the entire surface of concrete, the maximum stress of the L-A1 specimen with a small transverse reinforcement ratio exceeded the long-term allowable unit stress through stress increments due to corrosion expansion alone. The maximum stress of the L-A4 specimen with 4 times the transverse reinforcement ratio of L-A1 also increased rapidly to near the long-term allowable unit stress. Therefore, if the amount of corrosion corresponding to this step (corrosion penetration of 100 μm, cross-section loss of 2% or more) is reached, the structural performance of the RC member may be seriously affected. In the actual environment, as with Berra’s model [47], the amount of corrosion products entering the crack increases and the corrosion expansion pressure decreases according to the increase of crack width. Thus, additional examination considering the actual condition of RC is necessary for high-precision prediction. 

## 7. Conclusions

The aim of this study is to numerically investigate the effects of lateral confinement on the concrete cracking induced by corrosion of reinforced concrete. Based on the numerical results, the following conclusions can be drawn:

(1) In order to estimate the corrosion expansion rate of reinforcing bars, accelerated corrosion tests and finite element analysis using the corrosion expansion model by Lundgren for reinforced concrete specimens were conducted and compared. When the corrosion expansion rate of the reinforcing bar was set to 2, the analysis results (0.05–0.2 mm) approximately met the relationship between the crack width of concrete and corrosion rate of reinforcing bars (0.12 mm).
(2) In the range of 3–8 μm of corrosion depth, cracks developed independently in the concrete around the rebar, and the mechanical behavior of the bars, such as the corrosion–expansion relationship, were similar regardless of the position of the main reinforcement or the amount of transverse bars. However, above 10 μm of corrosion depth, the cracks became connected, and the crack behavior differed for each bar according to the position of the main reinforcement or the amount of transverse bars.
(3) For 2% corrosion rate of rebars, cracks exceeded the permissible width on the concrete surface, but they did not significantly affect the limit strength. Nevertheless, repair and reinforcement measures against cracks would be necessary because the promoting factors of neutralization or salting can reach the reinforcing bars through the connected cracks.
(4) Overall, the shear capacity of the reinforced concrete members will be affected by steel corrosion because the stress of the transverse bars reaches the yield strength at corrosion depths of 100–200 μm. However, a wider set of experimental data is required for accurate prediction because, as with the Berra model, the amount of corrosion particles flowing into the cracks increases and corrosion expansion pressure decreases with increasing crack width.

## Figures and Tables

**Table 1:** Composition and characteristics of concrete.

Component Materials (kg/m^3^)	
Water	175
Ordinary Portland cement (Type II)	350
Fine aggregate	780
Coarse aggregate (Maximum size: 20 mm)	968
Water/cement ratio (W/C)	0.5
Water reducer/cement (wt % by cement)	0.8
Average slump ^a^ (mm)	20.0
Average air content ^a^ (%)	4.6
Average compressive strength ^a^ (MPa)	42.5
Average splitting tensile strength ^a^ (MPa)	2.8
(performed 28 days after casting)	

^a^: Average over three tests.

**Table 2:** Corrosion expansion properties for each step.

Step	Expansion Pressure *p* (MPa)	Corrosion Expansion *e* (×10^−3^ mm)	Max. Crack Strain *ε_cr max_* (×10^−4^)	Corrosion Penetration *x* (×10^−3^ mm)	Cross-Section Loss *a*_loss_ (%)
8	4.0	1.66	0.03	5.3	0.11
19	9.5	6.00	0.27	27.3	0.53
27	11.5	8.95	4.74	45.5	0.95
29	11.7	10.00	5.52	51.4	1.07
30	11.8	26.60	18.30	141.0	2.94

**Table 3:** Details of specimens for numerical simulation.

ID	Loading Method	Cross-Sectional Area of Transverse Reinforcement
L-A0	Internal pressure control	0 mm^2^ (*p_w_* = 0)
L-A1	Internal pressure control	28 mm^2^ (*p_w_* = 0.47%)
L-A4	Internal pressure control	112 mm^2^ (*p_w_* = 1.87%)
